# Evaluating the Use of an Aerosol Box During Simulated Intubations

**DOI:** 10.7759/cureus.16507

**Published:** 2021-07-20

**Authors:** Andres de Lima, Michael J Chen, Aamir Abbas, Satya K Ramachandran, John D Mitchell

**Affiliations:** 1 Anesthesiology, Critical Care, and Pain Medicine, Beth Israel Deaconess Medical Center, Harvard Medical School, Boston, USA

**Keywords:** simulation, covid-19, intubation box, motion tracking, laryngoscopy

## Abstract

To evaluate the use of an aerosol box during video laryngoscopy intubation, we conducted a two-phase simulation-based study to assess if there were significant differences in time needed to safely intubate a patient with an aerosol box in place, as well as assess changes in laryngoscopists’ hand motions as determined by changes in accelerometry. 20 anesthesiology providers from our institution participated in the first phase assessing the time to intubation. Use of the aerosol box led to statistically significant increases in intubation times (Wilcoxon-Signed Rank test p < 0.001, z-score = 3.921), with the calculated Pearson’s correlation coefficient (r = 0.877) indicating a large effect size. An 8.5 - 11.5 second difference in median intubation times was maintained between corresponding attempts with versus without the aerosol box. 15 participants completed an optional post-assessment survey, with 10 of 15 respondents firmly stating they would not use the box in clinical practice. The hand accelerometry assessment included five anesthesiology providers from our institution. This revealed a statistically significant increase in trials with aerosol boxes for the left hand’s general accelerometry with a medium effect size (p = 0.031; z = -1.873; r = -0.484), as well as for the right hand’s general accelerometry with a large effect size (p < 0.001; z = -3.351; r = -0.865). Although the aerosol box is an interesting concept, its use is associated with increased time to intubation and a change in ergonomics, which may increase risk during airway management and represents a concern for patient safety.

## Introduction

The coronavirus COVID-19 pandemic, caused by Severe Acute Respiratory Syndrome Coronavirus-2 (SARS-CoV-2), created a unique set of challenges for healthcare systems. Inadequate access to personal protective equipment (PPE), particularly for healthcare providers in high-risk environments, resulted in the development of alternative barrier methods. An intubation aerosol box was initially described in Taiwan as a means to limit clinicians’ exposure to aerosolized particles when manipulating the airway [1]. Almost immediately after this report, aerosol boxes were being replicated, modified, and trialed for use by healthcare teams across the globe. A preliminary study demonstrated theoretical effectiveness in reducing exposure risk to aerosolized particles during intubation by protecting against potential secretions produced during airway management [2]. However, more recent studies have found that laryngoscopists using an aerosol box during intubation may, in fact, be at higher exposure risk to aerosolized particles [3] and that use of the aerosol box can even hinder intubation efforts [4-5].

To further evaluate the risk mitigation effect of an aerosol box during intubation, we conducted a two-phase simulation-based study at Beth Israel Deaconess Medical Center (BIDMC) in Boston, Massachusetts, United States. We sought to formally assess the impact of the aerosol box with respect to the time required to secure the airway during intubation as a surrogate measure for the safety of the device, as well as to assess changes in laryngoscopists’ hand motions as determined by changes in accelerometry [6]. The primary goals of the study were to determine if there were significant differences in time to intubation and hand motions when performing intubation on a mannequin with an aerosol box in place, compared to standard intubating conditions. Secondary goals included an assessment of provider feedback general sentiment on the use of an aerosol box during simulated intubations.

## Materials and methods

These studies were conducted under approval by BIDMC’s Institutional Review Board (IRB), the Committee on Clinical Investigation, as protocols #2020P000570 (Simulation-based Evaluation of Aerosol Box Use for Intubation of COVID Patients) and #2020P001064 (Simulation-based Assessment of Hand Accelerometry During Aerosol Box Intubation). Both protocols were approved with a waiver of documentation of informed consent. For both phases, participants provided prospective agreement to collect data on their intubation attempts, and data collection was done in a de-identified fashion. Aside from performance metrics during intubations, only participants’ level of training was recorded. Survey responses were optional and anonymous: neither identifiers nor participants’ level of training was captured as part of the survey. Survey responses were collected only from participants involved in studying the impact of the aerosol box on time to intubation. Statistical analyses were performed using Microsoft Excel (Version 16) and R [7-8], apart from the use of the corresponding calculator from Social Science Statistics to derive z-values for Wilcoxon-Sign Ranked tests [9].

**Figure 1:**
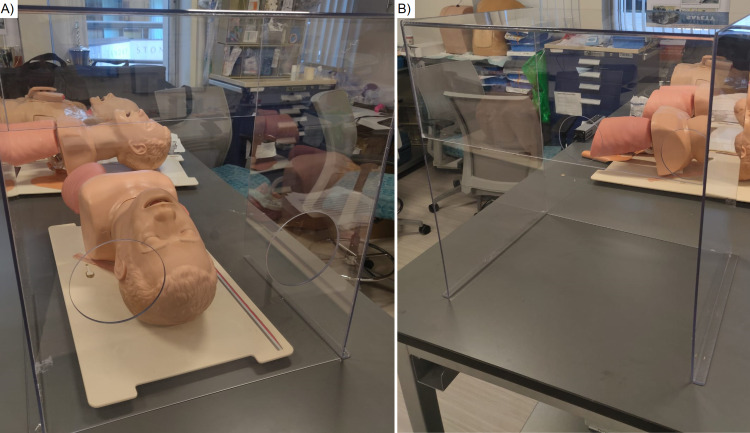
Aerosol box used in our study Figure 1A depicts the aerosol box placed over our intubation mannequin, with the intubator’s end of the box in the foreground. Figure 1B depicts the aerosol box by itself, with the intubator’s end of the box in the background.

The aerosol box was produced locally by P&S Machining and Fabrication (P & S Machine Co, Burlington, North Carolina) using clear polycarbonate plastic following the model previously described by Dr. Hsien Yung Lai and colleagues [1]. A high-fidelity intubation mannequin was used for all simulated intubations (Laerdal ® Airway Management Trainer, Laerdal, Norway). Figure 1 depicts the aerosol box placed over the mannequin. McGRATH^TM^ MAC Video Laryngoscopes (Medtronic plc, Dublin, Ireland) fitted with #3 blades were used to perform intubations with a 7.0 cuffed endotracheal tube (ETT) pre-loaded with a stylet. McGRATH^TM^ video laryngoscopes have been favored at our institution for intubation of COVID patients and are readily available at all practice locations for anesthesia providers. Participants did not wear personal protective equipment (PPE) during the simulated intubations in order to preserve PPE for clinical use.

Phase 1 - impact on time to intubation

Assessment on the impact of an aerosol box on time to intubation was performed with a convenience sample of twenty anesthesia providers, as anesthesiology providers were frequently preoccupied with providing clinical care in the context of the COVID-19 pandemic. Intubation attempts were recorded from May 5, 2020, to May 20, 2020. Each participant first performed five intubations without an aerosol box and then five intubations with the aerosol box in place, resulting in a total of ten intubations per participant. For intubations without the aerosol box, participants began hands-free, with the video laryngoscope and ETT next to the mannequin. For intubations performed with an aerosol box, participants' starting positions were as described above, with the addition of an aerosol box placed next to the head of the mannequin. When instructed to begin, participants were required to position the box over the head of the mannequin. Time to intubation was defined as the time between being instructed to start the intubation until the ETT cuff was inflated. Times were recorded with a stopwatch, rounded to the nearest whole second. There were no standardized breaks between intubations. Attempts without the box (1-5) always preceded attempts with the box (6-10).

Following the ten intubations, each participant was given the opportunity to complete an anonymous survey for feedback. Surveys were designed and deployed using Google Forms (Google LLC, California, United States) and consisted of five open-text questions regarding participants’ perceptions of the aerosol box. Survey responses were manually reviewed and collated by common themes.

A Shapiro-Wilk test determined that our data followed a non-parametric distribution. Therefore, we used a Wilcoxon Signed-Rank test to assess whether there were statistically significant differences in median intubation times without the aerosol box versus intubations with the aerosol box. To assess the effect size of differences in intubation times, we used the z-score from the Wilcoxon Signed-Rank test to calculate a Pearson correlation coefficient, r, using the formula “r = z /√ (N)” where z equals the z-score and N equals study size [10]. Kruskal-Wallis tests were used to assess whether there were statistically significant differences between participants grouped by level of training. A p-value of less than .05 was considered significant.

Phase 2 - assessment of hand motions per changes in accelerometry

To assess whether the presence of an aerosol box resulted in a significant impact on hand motions during intubation, we assessed accelerometry using motion sensors. Five additional anesthesia providers from February 10, 2021, to March 22, 2021: one in each class from the anesthesia residency program and an attending. These participants were unique to those recruited from phase 1 of this study and had no prior experience performing intubations with an aerosol box. Feedback was not solicited from participants during this phase of the study.

During this phase of our study, intubation instructions were similar to those described above. However, participants were only required to perform three video laryngoscopy intubations without the aerosol box in place, followed by three video laryngoscopy intubations with the aerosol box in place. Additionally, as the focus of this phase was the assessment of accelerometry, the aerosol box was already in place over the mannequin for the appropriate intubations. MetaMotionC (MBIENTLAB Inc., San Francisco, California) sensors were placed on the dorsum of participants’ left and right hands. The MetaMotionC sensors collect anonymous accelerometer data in the x, y, and z axes of each sensor every 0.08 seconds (12.5 Hz).

Initial accelerometry values were not always zero despite participants resting their hands on the table for the first few seconds of each trial; therefore, we manually zeroed values by subtracting the initial data value of an axis from each datapoint; an example of this process is depicted in Video 1, along with other calculations. This allowed us to account for any potential calibration errors affecting trials’ accelerometry values and subsequent analyses.

**Video 1:** Overview of Calculations In this video, author Michael Chen explains how various calculations using the sensor's accelerometry values were executed in a spreadsheet.

To assess general accelerometry of hands during intubations, we took the root mean square (RMS) of the x, y, and z axes’ accelerometry values for each time point in a trial. The tri-axial RMS values for each time point were then aggregated and divided by the trial duration, producing a time-adjusted value for aggregated RMS’s of tri-axial accelerometry values. This allowed us to assess whether general hand accelerometry significantly differed between trials with versus without aerosol boxes.

To visually compare the difference in hand accelerometry over time, the cumulative tri-axial RMS values for each time point were divided by the cumulative time of the trial up to their respective time point, then plotted on a graph’s y-axis. The x-axis of graphs corresponded to the percentage of a trial’s duration that had elapsed for each data point; these percentages were taken by dividing the time elapsed by the trial’s total duration. As this was an exploratory effort, and displaying graphs for each respective trial comparison in a meaningful way would not be feasible, we elected to showcase only one graph per participant comparing equivalent trials with and without aerosol boxes. As part of an interim analysis to visually compare hand accelerometry over time, we also compared corresponding simulated intubations with and without the box, with z-scores of raw, unadjusted tri-axial RMS values on the y-axis and trial completion percentage on the x-axis. Although we ultimately chose to use the formerly described graphical comparison, this method of graphing z-scores did highlight certain phases of increased activity during trials.

As part of a secondary analysis effort, we also assessed whether there were significant differences in hand accelerometry specific to individual axes for each hand. First, we took the RMS of all of a trial’s values in a single axis for each hand and axis; the RMS values were then divided by the total time spent during the trial to produce a time-adjusted single-axis RMS value. Time-adjusted single-axis RMS values of trials without aerosol boxes were compared to those of trials with aerosol boxes for each hand and axis. This allowed us to assess whether hand accelerometry significantly differed in individual axes of each hand when comparing intubations with versus aerosol boxes and to what degree. A member of the study team analyzed testing data to translate x, y, and z axes into directions with respect to the anatomical planes of a patient while an intubator’s hands would be rotated 90 degrees to manipulate intubation materials.

As in the initial investigation, Wilcoxon Signed-Rank tests were used to assess for statistically significant differences in median values of trials with versus without aerosol boxes. Effect sizes were again measured using z-scores to calculate a Pearson correlation coefficient. P-values of less than 0.05 were considered significant.

## Results

Phase 1 - impact on time to intubation 

Participants consisted of five attendings, eight residents, one fellow, and six certified registered nurse anesthetists (CRNA). For data analysis, the eight participating residents and single fellow were grouped together and classified as trainees. Of the 20 participants, 15 completed the survey, yielding a response rate of 75%.

**Figure 2:**
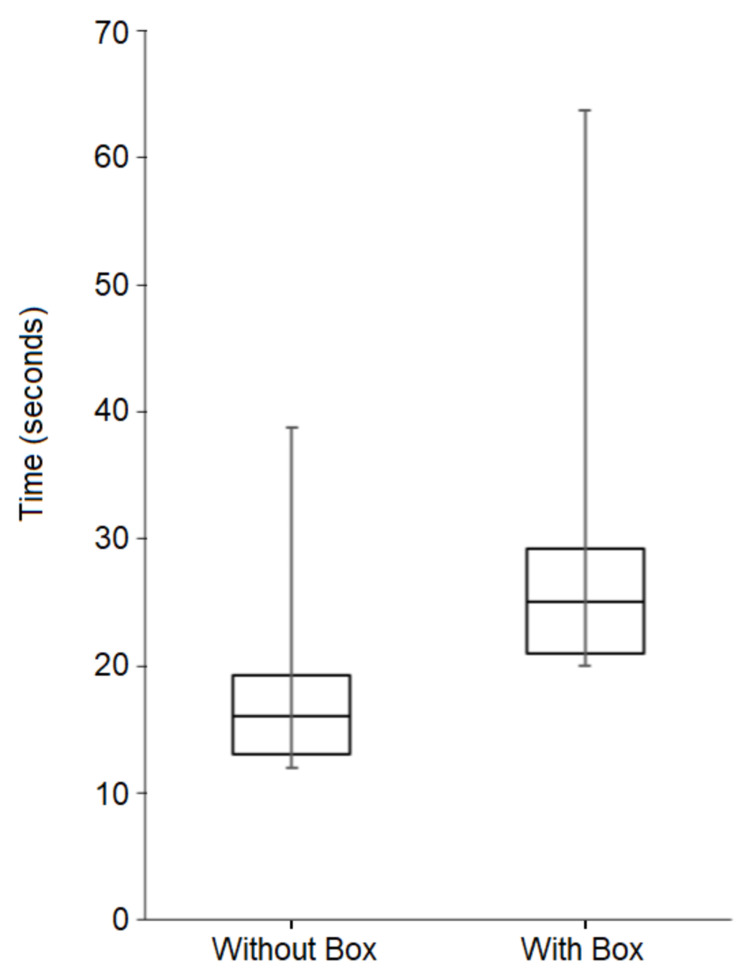
Box and whisker diagram summarizing intubation times for all attempts without the aerosol box versus the aerosol box Median and interquartile ranges (IQR) are depicted by the boxes, while whiskers represent extreme (maximum and minimum) values. The median time to intubation without the box was 16 seconds (IQR 13 to 19.25 seconds) and the median time to intubation with the box was 25 seconds (IQR of 21 to 29.25 seconds). This difference was statistically significant, as demonstrated by a Wilcoxon Signed-Rank test conducted for paired attempts with and without box, p < 0.001.

**Figure 3:**
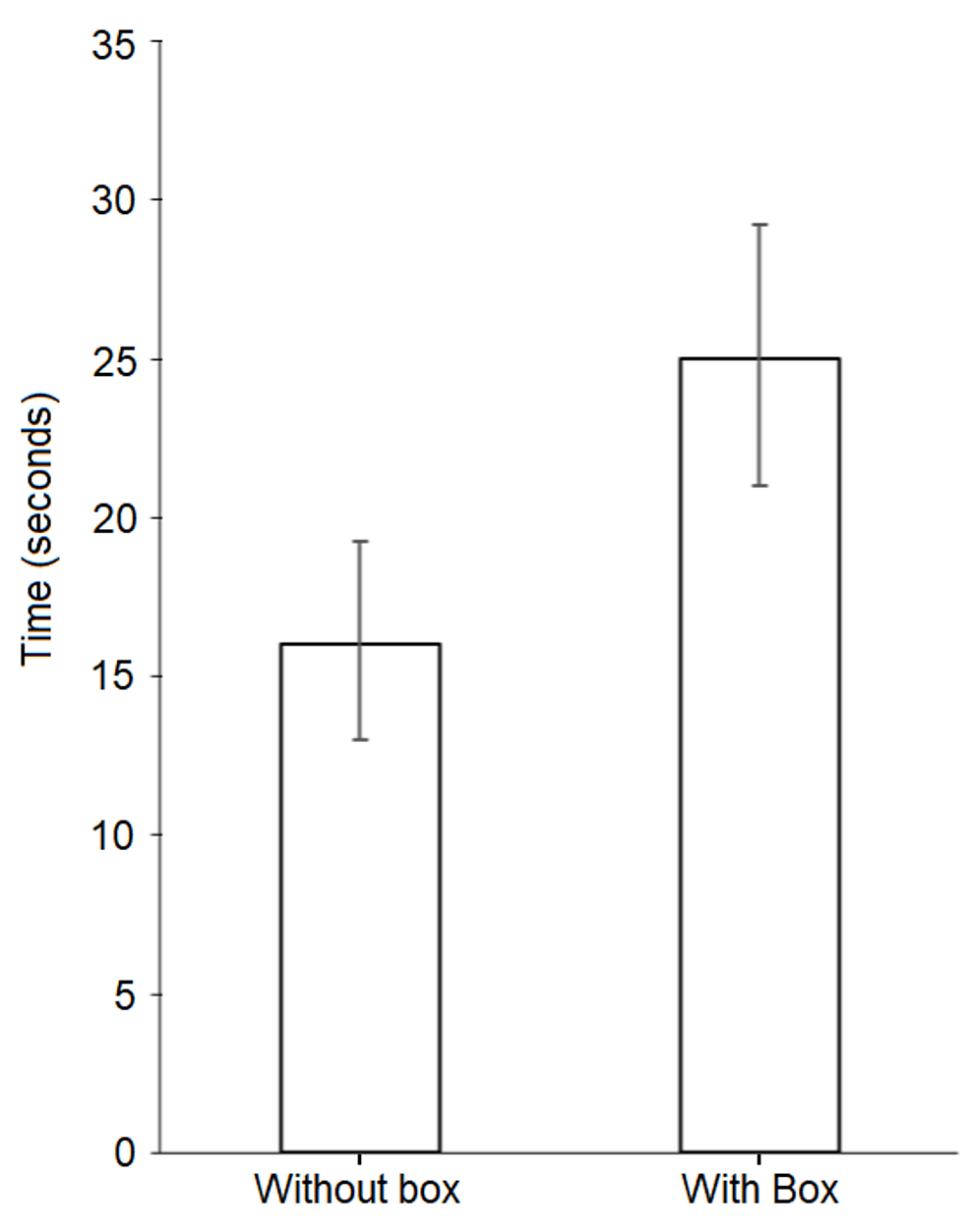
Column chart of median intubation times Columns depict median time to intubation for all attempts without the aerosol box versus all attempts with the aerosol box, with interquartile range (IQR) displayed as whiskers. The median time to intubation without the box was 16 seconds (IQR 13 to 19.25 seconds) and the median time to intubation with the box was 25 seconds (IQR of 21 to 29.25 seconds). This difference was statistically significant, as demonstrated by a Wilcoxon Signed-Rank test conducted for paired attempts with and without box, p < 0.001

Median time to intubation without the aerosol box was 16 seconds (interquartile range (IQR) 13 to 19.25 seconds), with times ranging from 9 to 42 seconds across all attempts without the box; each participant’s individual median times to intubation for their respective set of attempts without the box ranged from 10 to 24 seconds. For intubations with the aerosol box, median time to intubation was 25 seconds (IQR 21 to 29.25 seconds), with times ranging from 16 to 68 seconds across all attempts with the box; each participant’s individual median intubation times for their respective set of attempts with the box ranged from 18 to 37 seconds. Figures 2 and 3 depict median intubation times for all intubations with and without the aerosol box, with interquartile ranges. Table 1 displays median intubation times, as grouped by training level.

**Table 1:** Median intubation times with and without the aerosol box, grouped by training level All intubation attempts were completed on the first attempt without any complications. Trainees consisted of eight anesthesia residents and one anesthesia fellow. Statistically significant difference in intubation times between attempts with the aerosol box versus without the aerosol box (p < 0.001, z-score = -7.831). Pearson’s coefficient (r = -0.554) is indicative of a medium effect size. Differences between attendings, trainees, and certified registered nurse anesthetists (CRNA) were not found to be statistically significant for both median intubation times without the box (p = 0.832) and median intubation times with the box (p = 0.060) IQR - interquartile range

	Without Aerosol Box		With Aerosol Box	
Training Level	Median Intubation Time (s)	IQR (s)	Median Intubation Time (s)	IQR (s)
Attending (n=5)	16	14 - 22	27	23 - 35
Trainee (n=9)	15	14 - 19	24	20.25 - 27.75
CRNA (n=6)	15	13 - 18.75	25.5	23 - 29.75
All subjects (n=20)	16	13 - 19.25	25	21 - 29.25

A Shapiro-Wilk test determined that our data followed a non-parametric distribution (W = 0.877, p < 0.001). A Wilcoxon-Signed Rank test determined there was a statistically significant difference in intubation times between attempts with the aerosol box versus without the aerosol box (p < 0.001, z-score = -7.831). Using the z-score from the Wilcoxon-signed Rank test and the formula “correlation = z /√ (N)” we calculated a Pearson’s correlation coefficient (r) of -0.554, which indicated a medium effect size. Kruskal-Wallis tests determined there were no statistically significant differences between attendings, trainees, and CRNAs for both median intubation times without the box (p = 0.832) and median intubation times with the box (p = 0.060).

**Figure 4:**
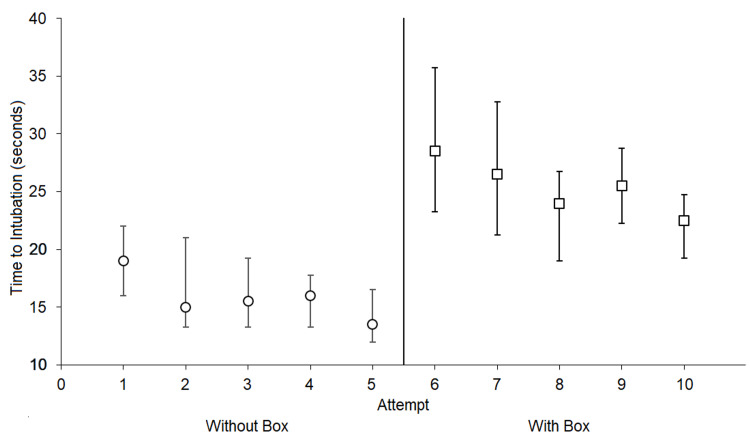
Median time to intubation for all participants per attempt trial Interquartile ranges (IQR) are displayed as whiskers. For all participants, intubation attempts 1 through 5 were without the aerosol box, followed by attempts 6 through 10 without the aerosol box. All intubation attempts were completed on the first attempt without any complications. A pattern for continuous improvement in times with increasing attempts suggests the presence of a learning curve.

Time to intubation improved over the course of each participant's set of five intubations, both with and without the aerosol box, as depicted in Figure 4. The median time to intubation for first attempts without the box was 19 seconds (IQR 16 to 22 seconds), with a median time for fifth attempts without the box of 13.5 seconds (IQR 12 to 16.5 seconds). With the aerosol box, the median time to intubation for first attempts was 28.5 seconds (IQR 21.25 to 33.75 seconds), with a median time for fifth attempts with the box of 22.5 seconds (IQR 20.25 to 25.75 seconds).

There were no occurrences of esophageal intubations or significant dental contact with the laryngoscope blade recorded in our study, both with and without the aerosol box. All participants were able to successfully intubate on their initial attempts, both with and without the aerosol box. We did not record time spent by participants positioning the aerosol box, although during practice attempts the authors report spending roughly five seconds positioning the box over multiple trials. 

Table 2 contains a summary of responses to each survey question, grouped by similar responses. Out of 15 respondents, only one reported they would use the box in clinical practice if clinically indicated for a patient with airborne infection to protect staff, ten respondents firmly stated they would not, and four respondents stated they would consider its use under specific conditions. 

**Table 2:** Summary of survey responses Responses were manually reviewed and collated by common themes. Several responses pertained to multiple themes. Response rate of 75% (n=15 out of 20 total participants); respondents responded to all questions.

Question	Summary of Responses and Identified Themes
Q1. What are your initial thoughts or impressions on the use of a barrier such as an acrylic box during intubation?	Cumbersome / restrictive (8 respondents)
	Potentially / conditionally useful (6 respondents)
	Increased difficulty for intubation (3 respondents)
	Concerns of concentrated / lingering aerosol after removal (2 respondents)
	Concern of false sense of reassurance with use of box (1 respondent)
Q2. Do you think you would use this in actual clinical practice? Why or why not?	Yes (1 respondent - if clinically indicated for a patient with airborne infection)
	No (10 respondents) Reasons:
	- Increased intubation difficulty (4 respondents)
	- Aerosolization is a non-issue with proper paralysis (3 respondents)
	- Cumbersome design (3 respondents)
	- Low confidence in conferred protective benefits (2 respondents)
	Conditionally (4 respondents)
	- With more practice using the box (1 respondent)
	- If modified (1 respondent)
	- If more information supported efficacy (1 respondent)
	- For extubation when coughing is more prominent (1 respondent)
Q3a. Do you think there are clinical scenarios in which these boxes or sheet barriers would be especially useful?	Yes - 10 respondents. Reasons:
	- Extubation (5 respondents)
	- Active coughing (3 respondents)
	- Routine cases (2 respondents)
	- Cases with aerosol/droplet precautions (1 respondent)
	- Emergency with others in proximity (1 respondent)
	Did not comment (5 respondents)
Q3b. Situations in which it would get in the way?	Yes (9 respondents) - Scenarios:
	- Difficult/emergent airways (4 respondents)
	- Obese patients (2 respondents)
	- Remote cases (1 respondent)
	Did not comment (6 respondents)
Q4. Did you feel use of the box hindered intubation performance? If so, how?	Yes (14 respondents)
	- Restricted movement (7 respondents)
	- Difficulty pulling stylet due to box height (4 respondents)
	- Visibility hindered (2 respondents)
	- Distracting (1 respondent)
	- Unable to suction (1 respondent)
	- Concern for obese patients (1 respondent)
	No (1 respondent, who only noted "Easy to use in routine intubations.")
Q5. What are improvements or changes you would suggest to the design or implementation of these barriers?	Provided suggestions - 12 respondents
	- Modify box's size (8 respondents)
	- Modify accessibility (8 respondents)
	- Modify visibility (4 respondents)
	- Modify weight (3 respondents)
	- Alternate methodology: "Keep it simple. Use ppe for intubation, sheet or towel for extubation." (1 respondent)
	No suggestions (2 respondents)

Phase 2 - assessment of hand motions per changes in accelerometry

For the hand accelerometry investigation, all five participants completed three simulated intubations without an aerosol box followed by three intubations with an aerosol box. As detailed in Table 3, Wilcoxon Signed-Rank tests comparing time-adjusted values for aggregate tri-axial RMS’s of accelerometry values revealed a statistically significant increase in trials with aerosol boxes for the left hand’s general accelerometry with a medium effect size (p = 0.031; z = -1.873; r = -0.484), as well as for the right hand’s general accelerometry with a large effect size (p < 0.001; z = -3.351; r = -0.865). 

**Figure 5:**
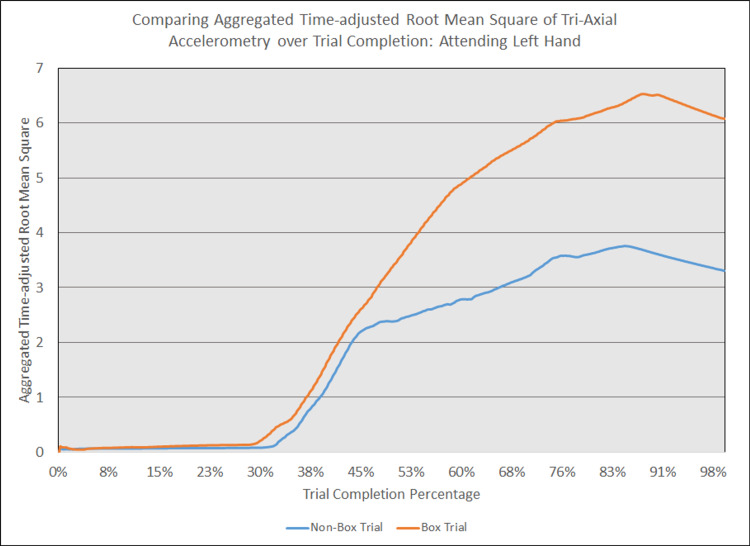
Comparing aggregated time-adjusted root mean square of tri-axial accelerometry over trial completion: attending left hand The above graph displays the aggregate general accelerometry measured in the left hand for an attending anesthesiologist's trial without an aerosol box, compared to that for their corresponding trial with an aerosol box. The y-axis measures the aggregated time-adjusted root mean squares of accelerometry values from all three axes' over a trial's duration. The x-axis measures the trial completion percentage, calculated by dividing the time elapsed at any given point by the respective trial's total trial duration. The blue line (bottom line at the end of the trial) depicts the trial without an aerosol box, while the orange line (top line at the end of the trial) depicts the corresponding trial with an aerosol box.

**Figure 6:**
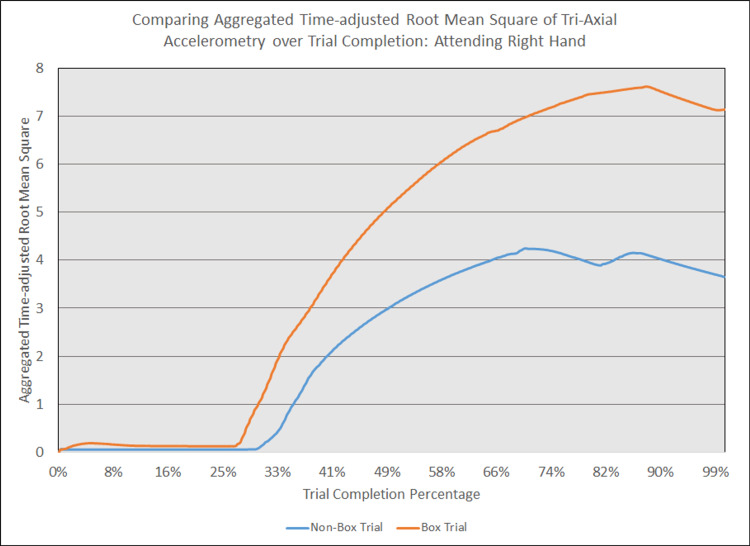
Comparing aggregated time-adjusted root mean square of tri-axial accelerometry over trial completion: attending right hand The above graph displays the aggregate general accelerometry measured in the right hand for an attending anesthesiologist's trial without an aerosol box, compared to that for their corresponding trial with an aerosol box. The y-axis measures the aggregated time-adjusted root mean squares of accelerometry values from all three axes' over a trial's duration. The x-axis measures the trial completion percentage, calculated by dividing the time elapsed at any given point by the respective trial's total trial duration. The blue line (bottom line at the end of the trial) depicts the trial without an aerosol box, while the orange line (top line at the end of the trial) depicts the corresponding trial with an aerosol box.

**Figure 7:**
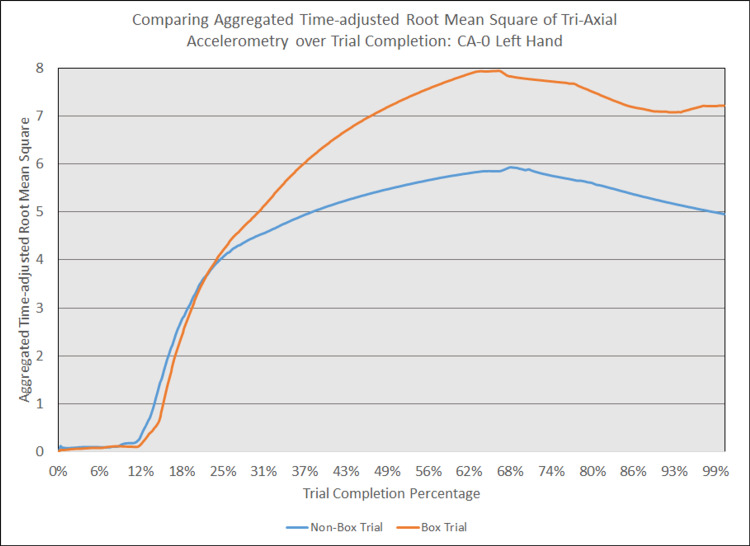
Comparing aggregated time-adjusted root mean square of tri-axial accelerometry over trial completion: CA-0 left hand The above graph displays the aggregate general accelerometry measured in the left hand for an intern in the anesthesiology residency program (CA-0)'s trial without an aerosol box, compared to that for their corresponding trial with an aerosol box. The y-axis measures the aggregated time-adjusted root mean squares of accelerometry values from all three axes' over a trial's duration. The x-axis measures the trial completion percentage, calculated by dividing the time elapsed at any given point by the respective trial's total trial duration. The blue line (bottom line at the end of the trial) depicts the trial without an aerosol box, while the orange line (top line at the end of the trial) depicts the corresponding trial with an aerosol box.

**Figure 8:**
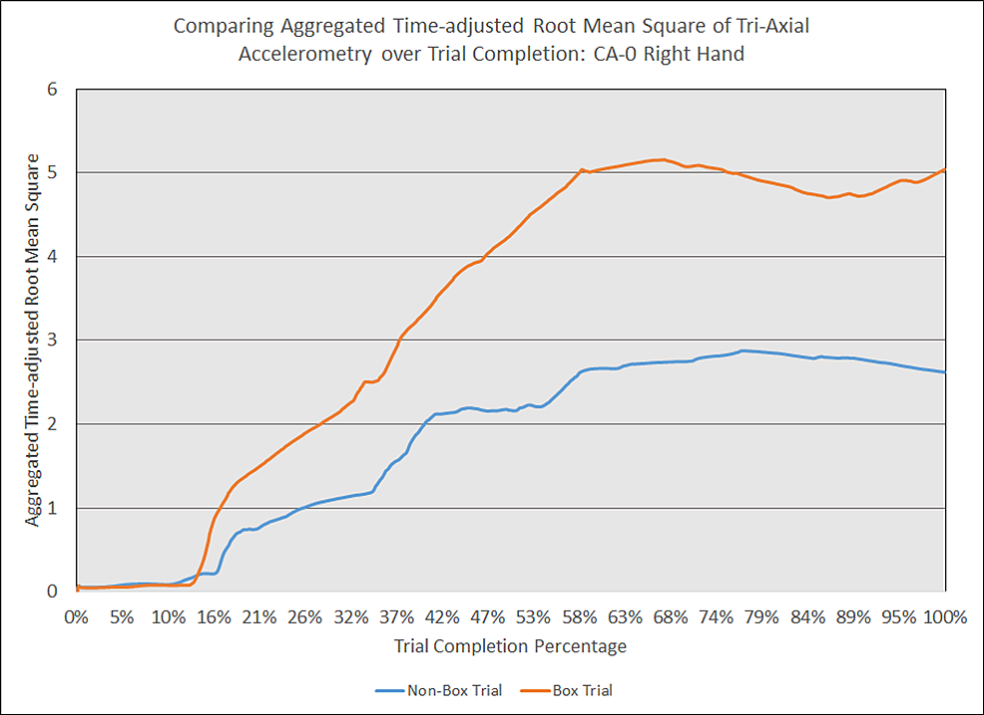
Comparing aggregated time-adjusted root mean square of tri-axial accelerometry over trial completion: CA-0 right hand The above graph displays the aggregate general accelerometry measured in the right hand for an intern in the anesthesiology residency program (CA-0)'s trial without an aerosol box, compared to that for their corresponding trial with an aerosol box. The y-axis measures the aggregated time-adjusted root mean squares of accelerometry values from all three axes' over a trial's duration. The x-axis measures the trial completion percentage, calculated by dividing the time elapsed at any given point by the respective trial's total trial duration. The blue line (bottom line at the end of the trial) depicts the trial without an aerosol box, while the orange line (top line at the end of the trial) depicts the corresponding trial with an aerosol box.

**Figure 9:**
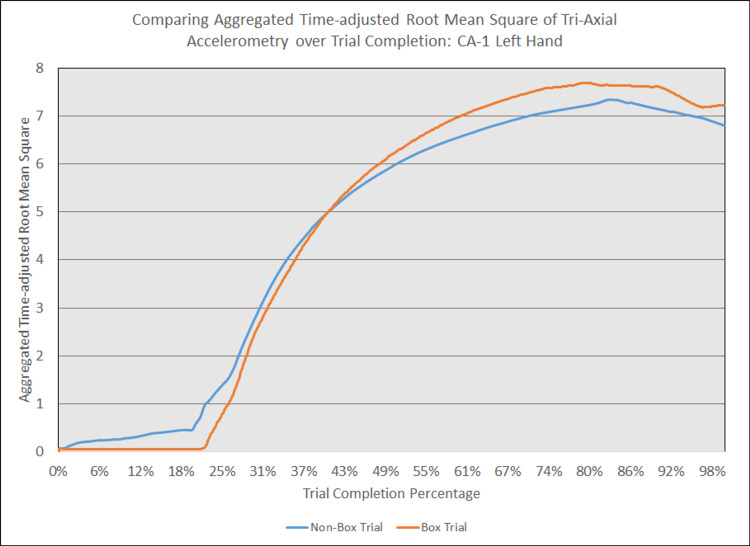
Comparing aggregated time-adjusted root mean square of tri-axial accelerometry over trial completion: CA-1 left hand The above graph displays the aggregate general accelerometry measured in the left hand for a first-year anesthesiology resident (CA-1)'s trial without an aerosol box, compared to that for their corresponding trial with an aerosol box. The y-axis measures the aggregated time-adjusted root mean squares of accelerometry values from all three axes' over a trial's duration. The x-axis measures the trial completion percentage, calculated by dividing the time elapsed at any given point by the respective trial's total trial duration. The blue line (bottom line at the end of the trial) depicts the trial without an aerosol box, while the orange line (top line at the end of the trial) depicts the corresponding trial with an aerosol box.

**Figure 10:**
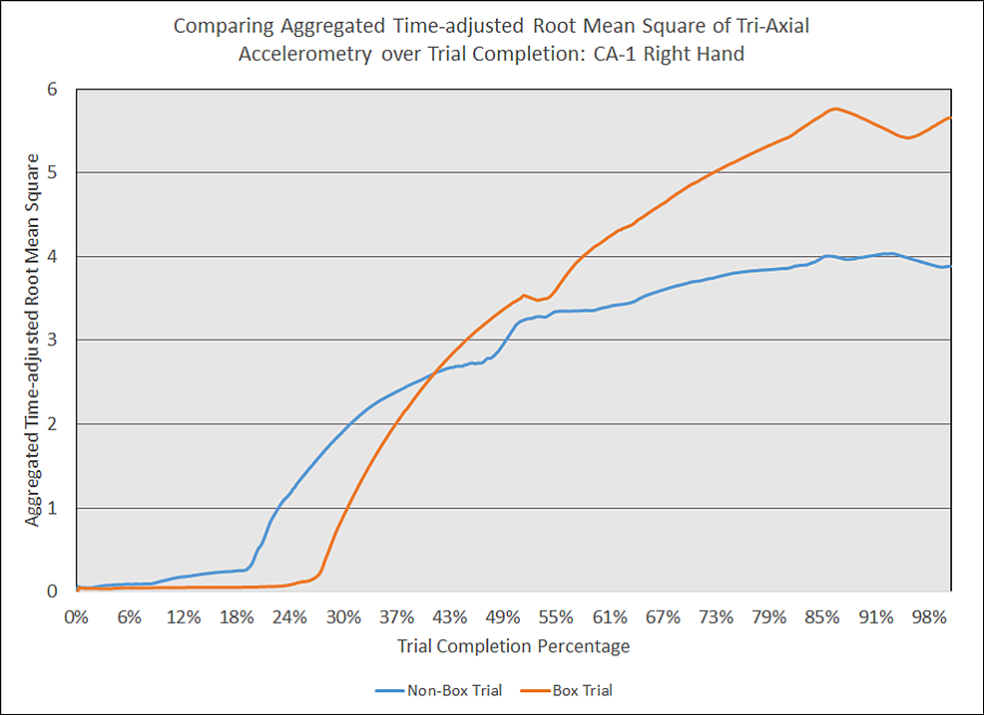
Comparing aggregated time-adjusted root mean square of tri-axial accelerometry over trial completion: CA-1 right hand The above graph displays the aggregate general accelerometry measured in the right hand for a first-year anesthesiology resident (CA-1)'s trial without an aerosol box, compared to that for their corresponding trial with an aerosol box. The y-axis measures the aggregated time-adjusted root mean squares of accelerometry values from all three axes' over a trial's duration. The x-axis measures the trial completion percentage, calculated by dividing the time elapsed at any given point by the respective trial's total trial duration. The blue line (bottom line at the end of the trial) depicts the trial without an aerosol box, while the orange line (top line at the end of the trial) depicts the corresponding trial with an aerosol box.

**Figure 11:**
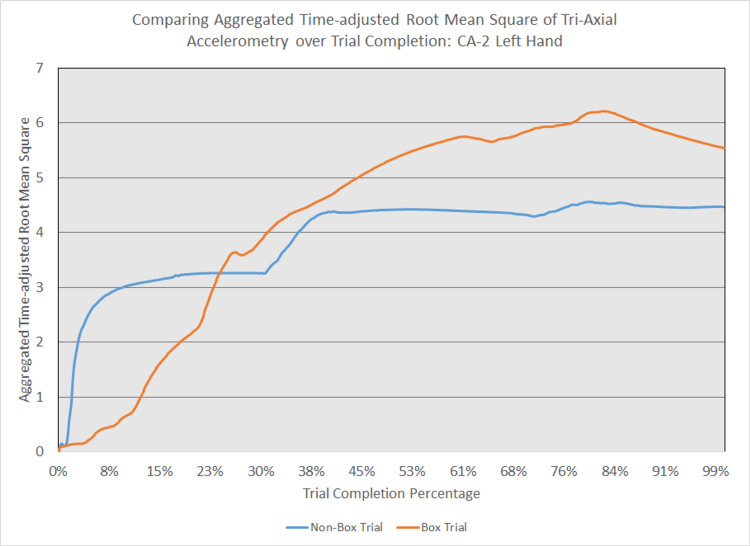
Comparing aggregated time-adjusted root mean square of tri-axial accelerometry over trial completion: CA-2 left hand The above graph displays the aggregate general accelerometry measured in the left hand for a second-year anesthesiology resident (CA-2)'s trial without an aerosol box, compared to that for their corresponding trial with an aerosol box. The y-axis measures the aggregated time-adjusted root mean squares of accelerometry values from all three axes' over a trial's duration. The x-axis measures the trial completion percentage, calculated by dividing the time elapsed at any given point by the respective trial's total trial duration. The blue line (bottom line at the end of the trial) depicts the trial without an aerosol box, while the orange line (top line at the end of the trial) depicts the corresponding trial with an aerosol box.

**Figure 12:**
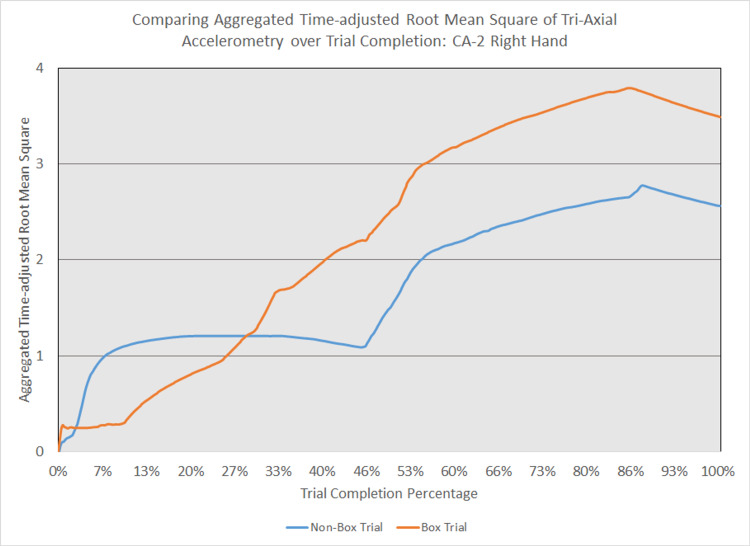
Comparing aggregated time-adjusted root mean square of tri-axial accelerometry over trial completion: CA-2 right hand The above graph displays the aggregate general accelerometry measured in the right hand for a second-year anesthesiology resident (CA-2)'s trial without an aerosol box, compared to that for their corresponding trial with an aerosol box. The y-axis measures the aggregated time-adjusted root mean squares of accelerometry values from all three axes' over a trial's duration. The x-axis measures the trial completion percentage, calculated by dividing the time elapsed at any given point by the respective trial's total trial duration. The blue line (bottom line at the end of the trial) depicts the trial without an aerosol box, while the orange line (top line at the end of the trial) depicts the corresponding trial with an aerosol box.

**Figure 13:**
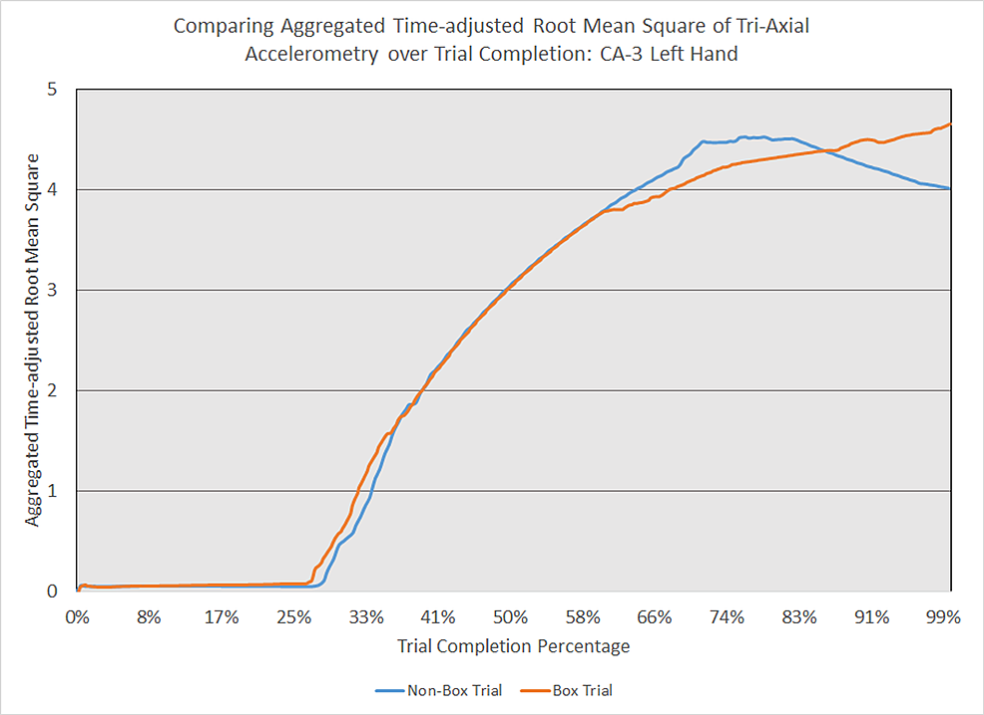
Comparing aggregated time-adjusted root mean square of tri-axial accelerometry over trial completion: CA-3 left hand The above graph displays the aggregate general accelerometry measured in the left hand for a third-year anesthesiology resident (CA-3)'s trial without an aerosol box, compared to that for their corresponding trial with an aerosol box. The y-axis measures the aggregated time-adjusted root mean squares of accelerometry values from all three axes' over a trial's duration. The x-axis measures the trial completion percentage, calculated by dividing the time elapsed at any given point by the respective trial's total trial duration. The blue line (bottom line at the end of the trial) depicts the trial without an aerosol box, while the orange line (top line at the end of the trial) depicts the corresponding trial with an aerosol box.

**Figure 14:**
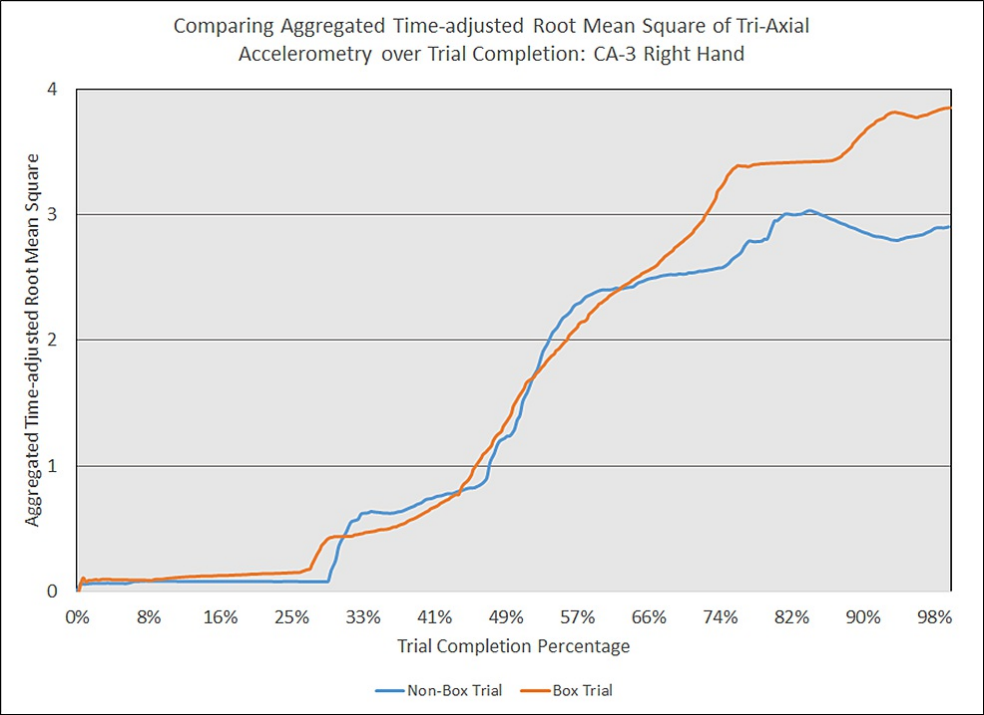
Comparing aggregated time-adjusted root mean square of tri-axial accelerometry over trial completion: CA-3 right hand The above graph displays the aggregate general accelerometry measured in the right hand for a third-year anesthesiology resident (CA-3)'s trial without an aerosol box, compared to that for their corresponding trial with an aerosol box. The y-axis measures the aggregated time-adjusted root mean squares of accelerometry values from all three axes' over a trial's duration. The x-axis measures the trial completion percentage, calculated by dividing the time elapsed at any given point by the respective trial's total trial duration. The blue line (bottom line at the end of the trial) depicts the trial without an aerosol box, while the orange line (top line at the end of the trial) depicts the corresponding trial with an aerosol box.

As depicted in Figures 5 - 14, while the general shape for graphs of corresponding intubation trials with and without aerosol boxes which chart the aggregate tri-axial RMS’s of accelerometry values over trial completion do align with one another, there are notable differences in time-adjusted aggregate tri-axial RMS’s values as the intubations progress. This was particularly apparent in the comparison of an attending’s third intubation trials with and without the aerosol box, as shown in Figures 5 and 6. 

**Figure 15:**
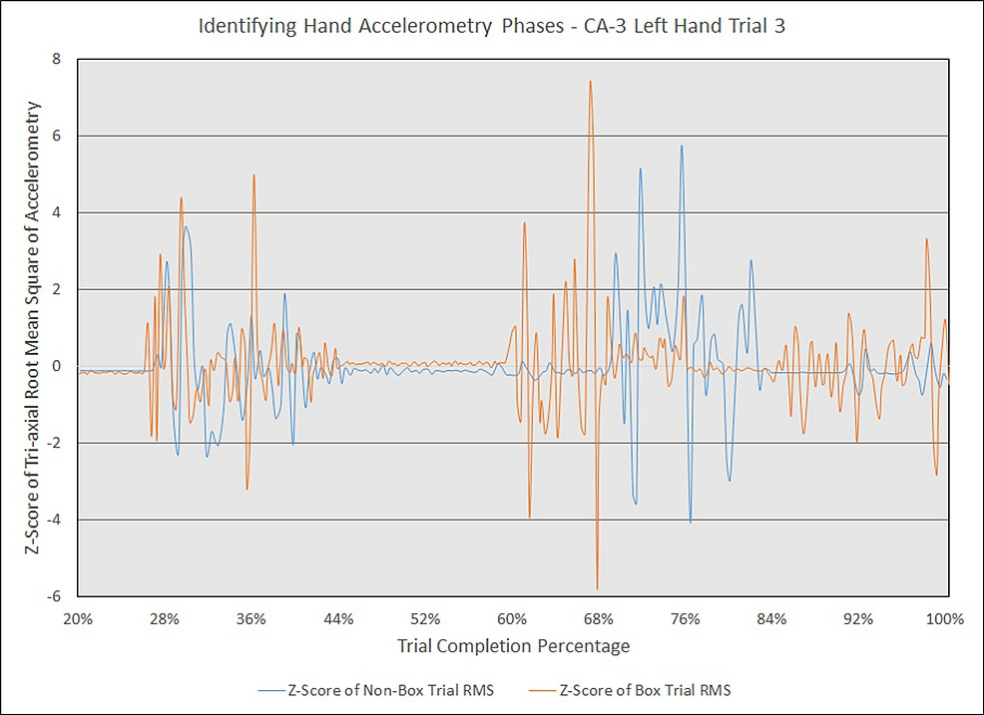
Identifying hand accelerometry phases - CA-3 left hand trial 3 The above graph highlights the phases at which general accelerometry peaked in the left hand for a third-year anesthesiology resident (CA-3)'s third trial without an aerosol box, compared to that for their corresponding third trial with an aerosol box. The y-axis measures the z-scores of the root mean square (RMS) of accelerometry values from all three axes' at a given point, with the z-scores derived from the mean and standard deviation of a respective trial. The x-axis measures the trial completion percentage, calculated by dividing the time elapsed at any given point by the respective trial's total trial duration. The x-axis starts at the 20% trial completion percentage, as values prior to that point are relatively stable with virtually no change in z-scores. This graph is notable for three phases of increased accelerometry, which begin around the 28% completion point, 60% completion point, and 84% completion point. Although the trials were not completely in phase with one another, the line representing the trial with the aerosol box (depicted in orange) is notable for having more prominent activity peaks than the line representing the trial without an aerosol box (depicted in blue).

**Figure 16:**
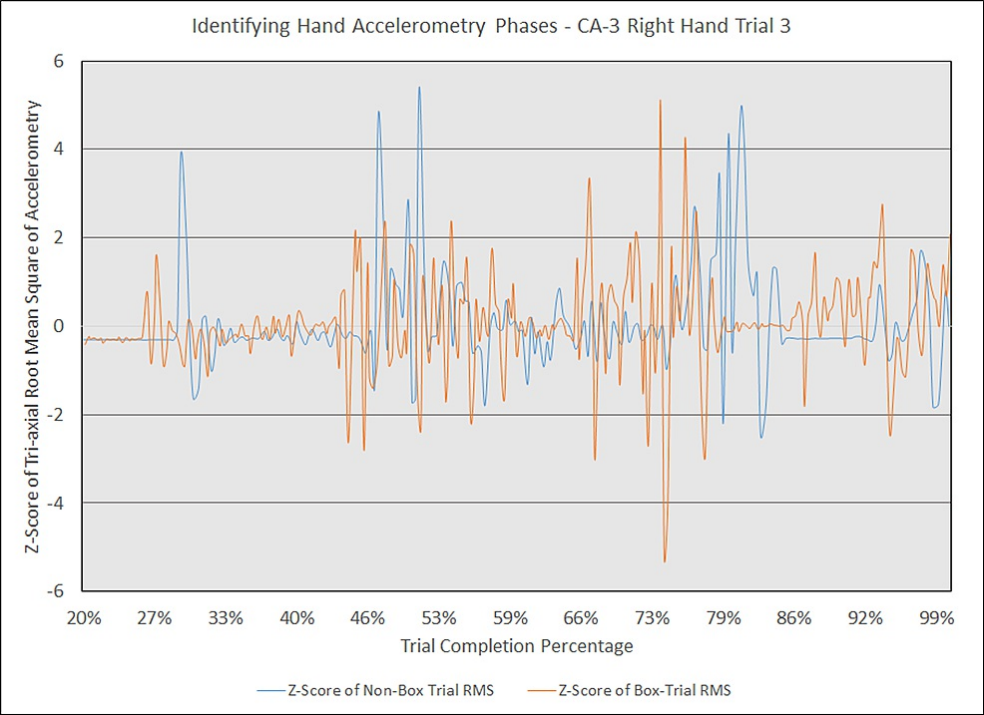
Identifying hand accelerometry phases - CA-3 right hand trial 3 The above graph highlights the phases at which general accelerometry peaked in the right hand for a third-year anesthesiology resident (CA-3)'s third trial without an aerosol box, compared to that for their corresponding third trial with an aerosol box. The y-axis measures the z-scores of the root mean square (RMS) of accelerometry values from all three axes' at a given point, with the z-scores derived from the mean and standard deviation of a respective trial. The x-axis measures the trial completion percentage, calculated by dividing the time elapsed at any given point by the respective trial's total trial duration. The x-axis starts at the 20% trial completion percentage, as values prior to that point are relatively stable with virtually no change in z-scores. This graph is notable for four phases of increased accelerometry which begin around the 27% completion point, 40% completion point, 66% completion point, and 86% completion point. Although the trials were not completely in phase with one another, the line representing the trial with the aerosol box (depicted in orange) is notable for having more prominent activity peaks than the line representing the trial without an aerosol box (depicted in blue).

Additionally, in an earlier attempt to graphically compare hand accelerometry over trial completion for corresponding trials, we observed that changes in accelerometry tended to cluster in three to four phases during trials with and without the box. Figure 15 displays an example of the three phases seen in the left hand for an attending physician’s corresponding simulated intubations with and without the box, with z-scores of raw, unadjusted tri-axial RMS values on the y-axis and trial completion percentage on the x-axis. Figure 16 displays an example of the four phases seen in the same trials for the right hand. While this approach highlights phases in which accelerometry changes, it was overall more difficult to interpret than a graphical comparison of aggregate tri-axial RMS’s due to the constantly overlapping nature of these graphs. 

After analyzing testing data, we corresponded the sensors’ x, y, and z axes with the superoinferior, anteroposterior, and mediolateral planes with respect to a patient’s anatomy, respectively. As detailed in Figure 4, Wilcoxon Signed-Rank tests comparing time-adjusted single-axis RMS values revealed statistically significant increases in trials with aerosol boxes with large effect sizes with respect to the following hand-plane combinations: left hand-superoinferior, left hand-mediolateral, right hand-superoinferior, and right hand-anteroposterior (all p values ≤ 0.008; all r values ≤ -0.689). Values for the left-hand-anteroposterior combination were not statistically significant (p-value of 0.139), nor were values for the right-hand mediolateral combination (p-value of 0.689). 

## Discussion

General findings and their significance

This study demonstrates that the use of an aerosol box may result in a significant impact on intubation conditions that lead to a significant delay in times required to secure the airway. Additionally, the presence of the airway box significantly impacts the provider’s hand motion when attempting to secure the airway. Delays in securing the airway and increased motion signals when performing laryngoscopy under optimized conditions in simulated scenarios raise concerns with regards to the safety profile of the aerosol box, particularly when used in high acuity clinical settings with variable conditions and concomitant use of PPE. These findings are in line with similar studies published recently by Begley et al. [4], as well as Sorbello et al. [5].

Impact on time to intubation

Although progressive improvement in intubation times was observed with repeated attempts, both with and without the aerosol box, an 8.5 - 11.5 second difference in median intubation was maintained between corresponding attempts (i.e., 1 & 6, 2 & 7, etc.). While it is possible that substantial amounts of practice may lead to intubation attempts with the aerosol box reaching acceptable times in comparison to intubation attempts without the box, the time spent reaching such a proficiency level may be more appropriately spent focusing on other safety measures. We advise that institutions seeking to use the aerosol box should only consider its use as an adjunct safety measure to PPE when there are no concerns of compromising the safety of patients and staff; our advisory is consistent with other anesthesiologists’ opinions on the aerosol box [11-12].

Qualitative results

Due to safety concerns, 2/3 of all respondents firmly stated they would not use the box in clinical practice, with only 1 of 15 respondents considering its use if clinically indicated for a patient with an airborne infection. The comments described in the survey suggest a significant number of concerns with intubating conditions and restrained motor capabilities, both of which can place stress on providers and therefore further inhibit users’ performance [13]. A recent 2019 systematic review of predictors of difficult intubation reported the overall proportion of patients having a difficult intubation as 10% [14]. This is high enough for us to have reasonable concerns that the small delays in intubation time observed in this study involving simulated, straightforward intubations would be worsened significantly in the clinical setting. Delays in intubation time place patients at higher risk for oxygen desaturation. This is of particular concern for patients with super morbid obesity, respiratory disease, and/or other factors associated with decreased pulmonary reserve [15]. Given the comorbidities and significant pulmonary and cardiovascular complications associated with the COVID-19 population, any delay seen in a simulated environment represents an important safety concern in a clinical scenario [16-19].

Assessment of hand motions per changes in accelerometry

Results also demonstrate that the activity of intubating may be described by phases of increased accelerometry, which may be interpreted as phases of increased hand motion or higher motor activity. Although our data does not include any video footage that helps correlate these increases in acceleration with moments during intubation, we hypothesize that these represent stages of increased activity. For the left hand, we believe the first phase represents the placement of the laryngoscope inside the mouth; the second phase represents motion required for exposure of the glottic opening; and the third phase represents the removal of the laryngoscope from the mouth followed by removal of the stylet. For the right hand, we believe the first phase represents opening the mouth with a scissoring technique, the second phase represents withdrawing the hand to obtain the ETT, the third phase represents guiding the ETT into the trachea, and the fourth phase represents accelerometry experienced while stabilizing the ETT as the stylet is being removed.

Increased accelerometry signals observed during any of the phases of the intubation naturally lead to increases in cumulative motion throughout the entire intubation sequence for trials performed with the box in place, as exemplified in Figures 5 and 6. This suggests the presence of the box requires increased maneuvering required to achieve an appropriate laryngoscopy view (left hand), with increased movement to insert an ETT in the mouth and through the glottis. Data obtained from the right hand also shows a substantial increase in accelerometry, which is likely due to increased effort and movement required to place the ETT into the mouth and down the trachea-even when a grade 1 view was achieved. It was not uncommon to see participants having trouble withdrawing the laryngoscope or removing the stylet when the aerosol box was in place, as the object represented a mechanical obstacle to the use of two hands. 

Strengths, limitations, and future directions

As previously mentioned, there are several studies evaluating the use of the aerosol box for intubations under different simulated scenarios [2-5, 11-12]. The results described in this study add to existing discussion regarding the use of barrier methods during aerosolizing procedures. Nevertheless, this data includes participants with different training backgrounds (i.e., CRNAs and trainees), further enhancing the body of evidence. Additionally, the structured qualitative evaluation conducted through the use of a questionnaire identifies specific concerns based on direct provider experience. Interestingly, a study by Ellison et al., which evaluated the use of an aerosol box in clinical use for emergent intubations, reported that the majority of practitioners who used the device found it easy to use, although it was rarely used despite being a readily available option [20].

Literature supports the use of commercial motion sensors as a cost-effective method for kinematic evaluations involving human performance [19], but there are currently only a handful of articles that have described investigations involving motion sensors during intubations [6, 21-24]. However, to our knowledge, this is the first study that makes use of accelerometry motion sensors as a surrogate for evaluating how endotracheal intubation technique differs when practitioners are placed under experimental conditions. Our technique has not been statistically validated, yet findings demonstrate that this tool may be useful in describing patterns of motion seen in clinical tasks that require some degree of technical skill. Moreover, this tool demonstrated to be useful in noting differences in intubation mechanics under different scenarios.

Future studies should continue to explore and validate the different techniques available to describe metrics for clinical tasks like intubations. Results from such studies may help design simulation scenarios and provide individualized feedback based on performance data. Yet, more data is required to fully understand the clinical significance of the difference in metrics (including accelerometry) when performing procedural skills such as intubating.

Limitations to our study findings include having providers position the box prior to intubation and adding this time as part of the total intubation time. This was done to better simulate the use of the aerosol box in a clinical setting, in which mask ventilation prior to intubation would preclude box placement and may complicate management, particularly when the clinician is securing the airway without assistance (as frequently indicated to limit exposure). Although the difference in intubation times would be reduced when accounting for this factor, this does raise an important safety concern. It is important to note that the few seconds of difference observed during this controlled, stress-free, and optimized simulation scenario could translate to clinically relevant adverse outcomes in settings with less optimal conditions. Additionally, both phases of the study utilized a convenience sample, as anesthesiology providers were frequently preoccupied with providing clinical care in the context of the COVID-19 pandemic.

## Conclusions

In conclusion, the use of an aerosol box during video laryngoscopy intubation is associated with risks such as increased time to intubation and a change in ergonomics, which may increase risk during airway management, and therefore represents a significant concern for patient safety. When planning the use of an aerosol box during intubation, considerations to impact on patient safety should be weighed against pre-existing guidelines such as securing the airway using rapid sequence induction with deep neuromuscular blockade and use of PPE.
